# Difficulty of MRI Based Identification of Lesion Age by Acute Infra-Tentorial Ischemic Stroke

**DOI:** 10.1371/journal.pone.0092868

**Published:** 2014-03-20

**Authors:** Florian Grosse-Dresselhaus, Ivana Galinovic, Kersten Villringer, Heinrich J. Audebert, Jochen B. Fiebach

**Affiliations:** Center for Stroke Research Berlin, Charité-Universitätsmedizin Berlin, Berlin, Germany; University of Manchester, United Kingdom

## Abstract

**Background:**

Systemic thrombolysis in acute ischemic stroke is restricted to the 4.5 h time window. Many patients are excluded from this treatment because symptom onset is unknown. Magnetic resonance imaging (MRI) studies have shown that stroke patients presenting with acute supra-tentorial diffusion-weighted imaging (DWI) lesions that do not have matching lesions on fluid attenuated inversion recovery (FLAIR) are likely to be within a 4.5 hour time window. This study examines the DWI-FLAIR mismatch in infra-tentorial stroke.

**Methods:**

This was a retrospectively conducted substudy of the “1000+” study; a prospective, single-center observational study (http://clinicaltrials.gov; NCT00715533). Fifty-six patients with infra-tentorial stroke confirmed by MRI and known symptom onset who underwent the scan within 24 h after symptom onset were analysed. Two neurologists blinded to clinical information separately rated the DWI lesion visibility on FLAIR. Lesion volume, relative signal intensities of DWI and relative apparent diffusion coefficient values were determined.

**Results:**

Regarding baseline characteristics our study population had a median age of 66 years, a median time from symptom onset to MRI of 616.5 minutes, a median NIHSS of 3 and a median DWI lesion volume of 0.26 ml. A negative FLAIR allocated patients to a time window under 4.5 h correctly with a sensitivity of 55% and a specificity of 61%, a positive predictive value of 44% and a negative predictive value of 71%. FLAIR positivity decreased with age (p = 0.018), and showed no significant correlation to lesion volume (p = 0.145).

**Conclusions:**

In our study the DWI-FLAIR-Mismatch does not help to reliably identify patients within 4.5 h of symptom onset in acute ischemic infra-tentorial stroke. Thus therapeutical decisions based on the DWI-FLAIR mismatch estimation of time from onset cannot be recommended in patients with infra-tentorial stroke.

## Introduction

Systemic thrombolysis in acute ischemic stroke is restricted to the 4.5 h time window [1]. The assessment of time of symptom onset by use of specific MRI eligibility criteria, such as the absence of well-developed fluid-attenuated inversion recovery (FLAIR) changes of acute diffusion-weighted imaging (DWI) lesions, is still under discussion [2]–[7]. However, most studies report a high predictive value of DWI-FLAIR mismatch to identify early stroke patients eligible for thrombolysis [3]–[7]. Based on these imaging findings patients with unknown symptom onset time might benefit from an emergency reperfusion therapy when showing a DWI-FLAIR mismatch [5]. In a study by Aoki et al. 50% of patients who were FLAIR negative past a 6-hour time window had an infra-tentorial lesion [7] therefore hinting at a difference in signal behaviour between supra- and infra-tentorial stroke. Independently, previous studies have reported that a false negative DWI result is also more common in patients with infra-tentorial strokes than in those with supra-tentorial lesions [8]. However the development of signal changes on FLAIR images exclusively in acute infra-tentorial stroke has not yet been a subject of examination. Hence, this study was conducted with the aim to examine FLAIR signal development purely in acute infra-tentorial DWI lesions and to investigate its potential of identifying infarction within the 4.5 h time window.

## Materials and Methods

This was a retrospectively conducted substudy of the “1000+” study; a prospective, single-center observational study (http://clinicaltrials.gov; NCT00715533). Written informed consent was obtained from all patients. This study and the consent procedure were approved by the ethics committees (Charité Ethikkommission, Ethikausschuss 4 am Campus Benjamin Franklin, Charitéplatz 1, 10177 Berlin; Institutional Review Board number EA4/026/08).

We included 56 consecutive patients who presented to our hospital between September 2008 and June 2011 with ischemic infra-tentorial stroke confirmed by MRI upon arrival. Patients were excluded when the time of symptom onset was unknown (64 patients) or the symptoms had occurred more than 24 h prior to the examination (3 patients). Only patients with symptomatic infra-tentorial lesions with clear onset were chosen. MRI studies were performed on a 3-T MRI scanner (Tim Trio; Siemens AG, Erlangen, Germany). The MRI sequence parameters were as follows: for DWI (TE = 93.1 ms, TR = 7600 ms, field of view = 230 mm, matrix 192×192, 2.5-mm slice thickness) and for FLAIR (TE = 100 ms, TR = 8000 ms, TI = 2370.5 ms, field of view = 220 mm, matrix 256×232, 5-mm slice thickness).

DWI and FLAIR images were evaluated separately by two experienced raters (FGD, IG) blinded to clinical information in terms of time from stroke onset to imaging, NIHSS and symptoms. FLAIR and DWI images were both made available. Raters judged only infra-tentorial lesions as FLAIR positive (DWI lesion visible on FLAIR) or FLAIR negative (DWI lesion not visible on FLAIR). Additional supra-tentorial lesions were ignored the same as subacute or older FLAIR lesions which did not overlap with acute DWI lesions. Finally, in cases of discrepancy, a consensus rating was performed.

The volume of DWI lesions was calculated using a free available software package (MRIcron, Version 11 Nov 2011). Considering the small DWI lesion size the delineation of regions of interest was made manually. For patients with disseminated infra-tentorial lesions more than one region of interest was drawn.

Accordingly the region of interest for the unaffected side was defined by mirroring the DWI lesion manually and possibly corrected to avoid areas of leukoaraiosis, contralateral stroke or cerebrospinal fluid.

Absolute and relative signal intensity (rSI) was calculated for all lesions on DWI and apparent diffusion coefficient (ADC). The rSI was calculated by dividing the SI in the pathological side by that in the normal side and designated as rDWI and rADC.

### Statistical analysis

Statistical analyses were performed with SPSS 20 (IBM) and Clinical Calculator 1 (VassarStats, 2001–2009). Medians and interquartile ranges describe the results. The Mann-Whitney-U test was used to assess differences between patient groups. To assess the correlation between time from stroke onset and rDWI, rADC and age we used a linear regression model.

## Results

Fifty-six data sets were included of which 26 patients had a brainstem stroke (46.4%), 28 patients had a cerebellar stroke (50%) and two patients had both (3.6%). All of our patients exhibited a positive DWI at time of arrival. For baseline characteristics see Table 1. The median Wahlund score was 4 (IQR 1 - 6), with only 5 patients (9%) having infra-tentorial white matter damage which did not overlap with the area of the acute stroke. The median DWI lesion volume was 0.25 ml, without a statistically significant difference between FLAIR negative and positive patients (p = 0.145). In terms of age FLAIR negative patients were significantly older than FLAIR positive patients (p = 0.018). There was no correlation between age and time from stroke onset (Spearman correlation coefficient −0.203, p = 0.133) as potential bias.

**Table 1:** Baseline Characteristics and Group Comparisons.

	All Patients	FLAIR-negative * (n = 25)	FLAIR-positive * (n = 31)	Group Comparison P Value †
Age, years, median (IQR)	66 (56.2–73.8)	69 (63.5–75.5)	59 (53–72)	0.018
Sex, female, no (%)	15 (27%)	7 (28%)	8 (26%)	1.000 ‡
Lacunar stroke, no (%)	18 (32%)	11 (44%)	7 (23%)	0,149 ‡
NIHSS, median (IQR)	3 (1–5)	2 (1–5)	3 (1–5)	0.954
Time to MRI, median (IQR)	616.5 (208–1036,8)	341 (153–887)	882 (249–1111)	0.059
DWI lesion volume, ml, median (IQR)	0.26 (0.14–1.0)	0.24 (0.1–0.49)	0.36 (0.14–4.01)	0.145
rADC values, median (IQR)	0.61 (0.53–0.78)	0.71 (0.58–0.83)	0.59 (0.52–0.64)	0.019
rDWI values, median (IQR)	1.76 (1.49–2.22)	1.59 (1.37–1.76)	2.04 (1.71–2.37)	< 0.01

*judged by two raters with knowledge of DWI. † Mann-Whitney *U* test.

‡ Fisher's exact test, two-tailed. NIHSS indicates National Institutes of Health Stroke Scale; rADC, relative apparent diffusion coefficient; rDWI, relative DWI.

FLAIR was rated as negative in 11 out of 20 (55%) patients <4.5 hours and in 14 of 36 (38.9%) patients >4.5 hours after onset. A negative FLAIR correctly identified patients within 4.5 hours of symptom onset with a specificity of 61% (95% CI 44–76%), a sensitivity of 55% (95% CI 32–76%), a positive predictive value (PPV) of 44% (95% CI 25–65%) and a negative predictive value (NPV) of 71% (95% CI 52–85%); see Table 2. For a subanalysis of these parameters in cerebellar and brainstem stroke separately also see Table 2. FLAIR positive patients showed only a trend towards prolonged time between symptom onset and MRI, without reaching statistical significance (p = 0.059). In a subgroup of 26 patients who qualified clinically for thrombolysis with a median NIHSS score 5 (IQR 4–6.75) the sensitivity for early time window of negative FLAIR was 58%. Specificity and PPV were 50%, NPV was 58% and accuracy 54%.

**Table 2:** Predictive values of DWI-FLAIR mismatch for the identification of patients within 4.5 h of symptom onset.

	Sensitivity (95% CI)	Specificity (95% CI)	PPV (95% CI)	NPV (95%CI)
All patients (n = 56)	55% (32–76%)	61% (44–76%)	44% (25–65%)	71% (52–85%)
Patients with brainstem stroke (n = 26)	83% (36–99%)	50% (28–72%)	33% (13–61%)	90% (57–99%)
Patients with cerebellar stroke (n = 28)	46% (20–74%)	73% (45–91%)	60% (27–86%)	61% (36–82%)

PPV: positive predictive value; NPV: negative predictive value.

FLAIR negative patients had a lower rDWI and higher rADC value than FLAIR positive patients (p<0.05). In a linear regression model we could only show a weak correlation between delay from stroke onset and rDWI values (Spearman correlation coefficient 0.335, p = 0.012) and no correlation between delay and rADC values (Spearman correlation coefficient −0.36, p = 0.791).

## Discussion

**Figure 1:**
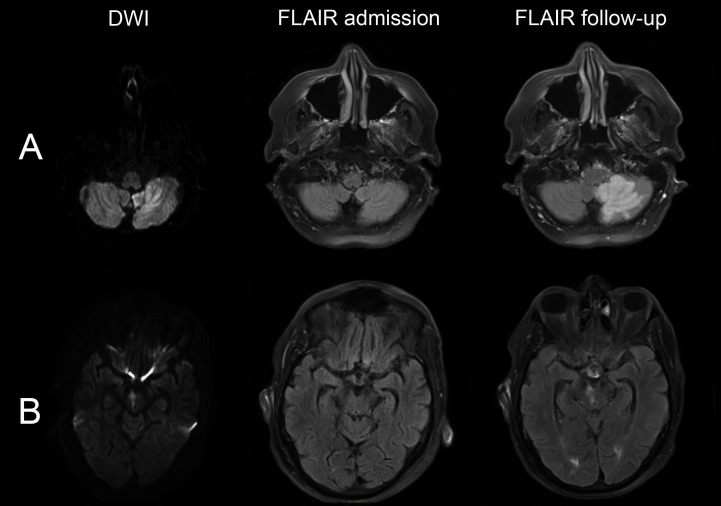
Examples of late FLAIR changes of acute infra-tentorial DWI lesions. The DWI shows a left cerebellar lesion (A) and a right mesencephal lesion (B) 22 h and 16,5 h after symptom onset, respectively. Both have no clear FLAIR visibility on the day of admission but are demarcated on the follow-up examination.

**Figure 2:**
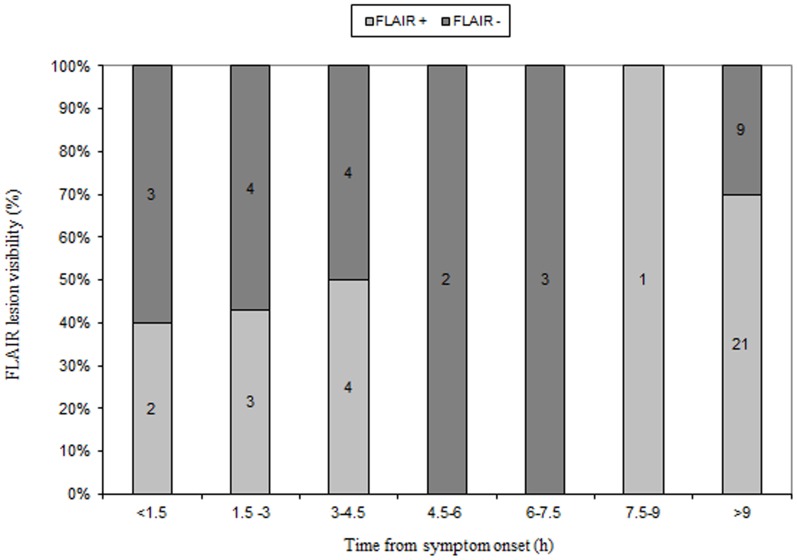
Visibility of FLAIR lesions and time from symptom onset. Numbers in the columns represent patients within each time interval.

Our study demonstrates the difficulty of estimating lesion age in infra-tentorial stroke. In this population, the DWI-FLAIR-Mismatch does not seem to reliably identify patients within 4.5 hours of symptom onset (Figure 1). Twelve patients were still FLAIR negative after 6 hours and two patients even after 20 hours. A total of 39% of patients with symptom onset > 4.5 hours were FLAIR negative (Figure 2). Three single-centre and one multi-centre studies have reported a time dependency of FLAIR visibility of acute ischemic lesions [2]–[4], [6]. Thomalla et al. examined in their single-centre study (<3 h) and multi-centre-study (<4.5 h) predominantly supra-tentorial strokes. They reported a high specificity (93% and 78%) and positive predictive value (94% and 83%) whereas sensitivity (48% and 62%) and negative predictive value (43% and 54%) were low [3], [4]. In our study specificity and positive predictive value were lower, sensitivity was comparably low, only the negative predictive value was higher. Based on our results of a higher negative and lower positive predictive value the change to FLAIR positivity would appear to take longer in infra-tentorial than in supra-tentorial stroke. This seems to be especially true for brainstem stroke (Table 2). Due to safety reasons, precisely PPV and specificity are important parameters in decision making regarding thrombolysis in acute stroke of unknown onset.

rDWI and rADC seem to evolve parallel to FLAIR, however the time dependency of rDWI was too weak for clinical use within a 24 h period. Corresponding to the observations of Ebinger et al. rADC values in our study did not chronometer stroke onset in a useful manner although it has been shown that ADC values decrease in the first three days of stroke [2], [9].

Finally FLAIR-positive patients were younger than FLAIR-negative patients. This observation was already made by Thomalla et al. for supra-tentorial stroke and shows that there are other factors influencing the development of FLAIR changes than time alone [4].

This study has several limitations such as the low number of patients in general and the limited number of patients per time window (e.g. only 20 patients in a time window <4,5 h). On average the patients had mild neurological deficits (median NIHSS 3) and small lesions (median 0.26 ml). Therefore this cohort was not representative of patients eligible for thrombolysis and may also explain lower predictive values of FLAIR compared to the cited studies. Furthermore the small DWI lesion volume might limit the accuracy of the quantification of diffusion parameters in some patients.

## Conclusion

In our study the DWI-FLAIR-Mismatch did not help to reliably identify patients within 4.5 h of symptom onset in acute ischemic infra-tentorial stroke. The importance of this study is in showing that the DWI-FLAIR mismatch in acute ischemic infra-tentorial stroke does not seem to be as helpful in identify patients within 4.5 h of symptom onset as it is in cases of supra-tentorial stroke. Thus, therapeutical decisions based on the DWI-FLAIR mismatch estimation of time cannot be readily recommended in patients with infra-tentorial stroke. This is of special relevance considering currently ongoing MRI based clinical studies examining the effect of thrombolysis in patients with unknown time of stroke onset.

On the other hand, and assuming that FLAIR may be a tissue clock, our study could further support the hypothesis that infra-tentorial ischemic stroke may benefit from late endovascular therapy independently from a rigid time window [10].
